# An Antibacterial-Loaded PLA 3D-Printed Model for Temporary Prosthesis in Arthroplasty Infections: Evaluation of the Impact of Layer Thickness on the Mechanical Strength of a Construct and Drug Release

**DOI:** 10.3390/pharmaceutics16091151

**Published:** 2024-08-30

**Authors:** Carlos Tamarit-Martínez, Lucía Bernat-Just, Carlos Bueno-López, Adrián M. Alambiaga-Caravaca, Virginia Merino, Alicia López-Castellano, Vicent Rodilla

**Affiliations:** 1 Instituto de Ciencias Biomédicas, Departamento de Farmacia, Facultad de Ciencias de la Salud, Universidad Cardenal Herrera-CEU, CEU Universities, C/Santiago Ramón y Cajal s/n, 46115 Alfara del Patriarca, Valencia, Spain; car.tamarit.ce@ceindo.ceu.es (C.T.-M.); lucia.bernatjust@uchceu.es (L.B.-J.); car.bueno.ce@ceindo.ceu.es (C.B.-L.); alopez@uchceu.es (A.L.-C.); 2 Departamento de Farmacia y Tecnología Farmacéutica y Parasitología, Facultad de Farmacia, Universitat de València, Av. Vicente Andrés Estellés s/n, 46100 Burjassot, Valencia, Spain; virginia.merino@uv.es; 3 Instituto Interuniversitario de Investigación de Reconocimiento Molecular y Desarrollo Tecnológico (IDM), Universitat Politècnica de València, Universitat de València, 46022 València, Valencia, Spain

**Keywords:** 3D printing, fused deposition modeling (FDM), printing layer thickness, controlled drug release, personalized prosthesis, polylactic acid (PLA), cloxacillin, vancomycin

## Abstract

Infections are one of the main complications in arthroplasties. These infections are difficult to treat because the bacteria responsible for them settle in the prosthesis and form a biofilm that does not allow antimicrobials to reach the infected area. This study is part of a research project aimed at developing 3D-printed spacers (temporary prostheses) capable of incorporating antibacterials for the personalized treatment of arthroplasty infections. The main objective of this research was to analyze the impact of the layer thickness of 3D-printed constructs based on polylactic acid (PLA) for improved treatment of infections in arthroplasty. The focus is on the following parameters: resistance, morphology, drug release, and the effect of antibacterials incorporated in the printed temporary prostheses. The resistance studies revealed that the design and layer thickness of a printed spacer have an influence on its resistance properties. The thickness of the layer used in printing affects the amount of methylene blue (used as a model drug) that is released. Increasing layer thickness leads to a greater release of the drug from the spacer, probably as a result of higher porosity. To evaluate antibacterial release, cloxacillin and vancomycin were incorporated into the constructs. When incorporated into the 3D construct, both antibacterials were released, as evidenced by the growth inhibition of *Staphylococcus aureus*. In conclusion, preliminary results indicate that the layer thickness during the three-dimensional (3D) printing process of the spacer plays a significant role in drug release.

## 1. Introduction

Prosthetic joint infection (PJI) is a formidable challenge in the field of arthroplasty [1]. Its prevalence ranges from 0.5% to 3%, and its repercussions include an escalating burden of comorbidity and mortality [2]. PJI can manifest acutely or chronically over the years, owing to the formation of persistent biofilms. Biofilms offer bacteria a protective haven, rendering infections resistant to treatment [3]. A biofilm is formed by the aggregation of microorganisms on the surface of the prostheses, where the bacterial colony becomes encased in a complex extracellular matrix, providing a shield against antimicrobials. This protective mechanism hinders the effectiveness of antibacterial treatment, contributing to the persistence of the infection over time [4]. The bacteria responsible for prosthesis infection are predominantly *Staphylococcus aureus* (26.5%), followed by coagulase-negative staphylococci (14.3%), Gram-negative microorganisms (8.2%), streptococci (4.4%), and anaerobes (3.4%) [5,6]. In addition, PJI is associated with symptoms such as local redness, warmth to touch, swelling, or joint stiffness and pain, with the appearance of wound leakage [7]. Microbiological diagnosis is made by culture of synovial fluid aspirate or periprosthetic tissue, and the treatment protocol includes debridement, administration of antibacterials, and implant retention surgery [8,9]. Several studies suggest combining systemic and local antibacterial prophylaxis, the latter by incorporating antibacterials into bone cements [10,11]. This methodology involves both topical and systemic antibacterial action [1,12]. Antibacterial-loaded bone cement (ALBC), particularly polymethylmethacrylate (PMMA) bone cement, has gained widespread usage in orthopedics for antibacterial treatment [13]. The patient’s knee joint needs to be kept free of any infected foreign prosthetic material during eradication of the infection. In addition, experts agree that some level of joint stability needs to be maintained, often with the help of a spacer [14]. Chemical stability and bactericidal properties, even at low concentrations, are desirable characteristics of antibacterials, which should not cause allergic reactions or facilitate the growth of resistant microbes. Tobramycin, gentamycin, vancomycin, and cephalosporines are the most popular antibacterials used for cement impregnation [15,16,17]. The use of ALBC has been linked to antibacterial resistance after repeated use of antibacterial-containing cements, which makes subsequent treatment, if required, more challenging [18,19]. Additionally, the compressive and flexural strengths of today’s bone cements are still rather low and, in some cases, insufficient to adequately stabilize the prosthesis [20].

Advances in three-dimensional (3D) printing offer a promising avenue for producing biomedical and pharmaceutical products. It involves the building up of multiple layers from a previously designed model that allows to build personalized structures [21]. The most common printing techniques are fused deposition modeling (FDM), ink-jet printing, and stereolithography (SLA) [22,23]. In FDM, a thermoplastic polymer is heated until it reaches a liquid state to be extruded and ejected into a printer bed, layer by layer. Each layer connects to the others below and solidifies as it cools [24]. The selection of suitable polymers is necessary to obtain an optimal printed material. Polylactic acid (PLA) has proved to be one of the strongest and most biocompatible materials using the FDM printing method. It is an FDA-approved polymer that is listed as safe and biodegradable [25]. PLA impregnated with antibacterials has been used and has shown promising antibacterial effects [26,27].

3D printing can be customized and fine-tuned by modifying printing parameters, such as layer thickness, filling percentage, and thermal processing parameters [28,29,30]. The vertical axis (*z*-axis) divides the printing layer’s thickness into segments. When the layer thickness decreases, the number of layers increases to achieve the printed structure. This leads to a higher *z*-axis resolution. In 3D printing, the layer thickness parameter is critical because it has an immediate effect on print quality, resolution, and printing time [31]. Using a higher layer thickness can speed up printing but may reduce the precision and quality of the finished product [32,33,34]. In addition, a lower layer thickness implies printing more layers. Since the layers are thinner, a lower layer thickness results in finer details and a smoother surface finish. This is significant because it allows for a higher level of accuracy when printing intricate designs or objects with complex geometries. As a result, choosing the right layer thickness requires striking a balance between print speed and resolution. The strength and durability of the printed object may increase as the layer thickness is reduced due to improved layer bonding [35]. This customization capability is particularly relevant for designing spacers that not only support the joint during the treatment of the infection but also deliver therapeutic agents directly to the infection site. Thus, understanding how variations in layer thickness affect the release of antibacterials from 3D-printed constructs is essential for optimizing their design and functionality. The main goal of this research was to investigate how varying the layer thickness and the predefined design of a 3D-printed PLA spacer affects the release of antibacterials for knee arthroplasty. To accomplish this objective, we conducted three separate studies using different print layer thicknesses (0.2, 0.3, and 0.4 mm): one focused on physical characterization, another on in vitro release assessments, and the third on microbiological studies.

## 2. Materials and Methods 

### 2.1. Materials

The PLA polymer used in this study was purchased from BQ^®^ (Huesca, Spain), while methylene blue (MB) was obtained from Guinama (Valencia, Spain). Cloxacillin (CLOX) and vancomycin (VAN) were acquired from Sigma Aldrich Chemical Co. (St. Louis, MO, USA), and Mueller Hinton medium broth and agar from Scharlab (Barcelona, Spain). Phosphate buffered solution (PBS) was composed of disodium hydrogen phosphate, sodium dihydrogen phosphate, sodium chloride, and bi-distilled water, all of which were supplied by Sigma Aldrich Chemical Co. (St. Louis, MO, USA). The pH of this solution was adjusted to 7.4 ± 0.1 by adding 5 N hydrochloric acid or 5 N sodium hydroxide, as required.

### 2.2. Design and 3D Printing 

**Figure 1:**
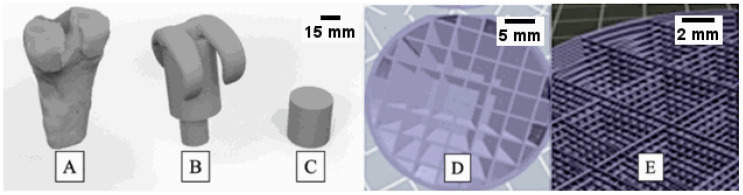
Digitalized femur (**A**) from human femur measurements, spacer (**B**) intended to be placed in the knee during PJI, and designed test construct (**C**) intended to contain antibacterials and spacer fill pattern of cross-linked overlapping beams (**D**,**E**) using “Rhinoceros 3D” software [36].

The process of design and 3D printing is depicted in Figure 1. To create the 3D-printed structure, a femur was first printed (Figure 1A). The diameters of several male and female human femurs were measured at our university’s anatomy department. The average measurements were found to be 33.29 ± 1.55 mm for males and 28.48 ± 0.57 mm for females. Subsequently, a spacer that could resemble these dimensions was designed (Figure 1B). Finally, a structure that could be inserted into the spacer was developed, and for this reason, a cylinder form was selected (Figure 1C). This cylindrical structure is intended to store antibacterials and provide controlled release during PJI. Different filling patterns were first tested until an optimal design was achieved. The resulting design consisted of a cylinder-shaped spacer with an interior pattern of crisscrossed overlapping beams and a 10% infill percentage (Figure 1D,E). The external volume of the cylinder was 23.12 cm^3^, closely resembling the dimensions of a human femur [36]. However, a smaller cylinder (1.20 cm^3^) was also designed and printed to carry out antibacterial release studies. Physical characterizations were carried out with both cylinders, whereas in vitro release and microbiological experiments were conducted only with the 1.20 cm^3^ structure.

The chosen printing polymer was PLA. Previous studies demonstrated that PLA exhibits superior strength and biocompatibility compared to other polymers such as polypropylene (PP), polyethylene terephthalate glycol (PET-G), and acrylonitrile butadiene stirene (ABS) plastic when using the FDM printing method [36,37,38]. 

To fabricate the 3D-printed construct used in this study, PLA filaments (BQ^®^, Huesca, Spain) were used with a standard fused-deposition modeling (FDM) 3D printer, Flashforge Creator Pro (Zhejiang Flashforge 3D Technology Co., Jinhua, China). The design of the printing template was created using Rhinoceros 3D software and exported as an FPP file (.fpp). Table 1 provides the main printing parameters of the Flashforge Creator Pro printer, which include the print and platform temperatures, print speed, infill percentage, and the different layer thicknesses tested in this study: 0.2, 0.3, and 0.4 mm. 

### 2.3. Physical Characterizations

The physical characterization of PLA 3D-printed constructs includes two main tests: resistance and morphology studies using model constructs in two sizes, namely 1.20 cm^3^ and 23.12 cm^3^.

The compressive strength test was carried out to determine the potential influence of layer thickness in the printed model on the mechanical properties of the structure. The point at which each layer superimposes the next was considered in the printing process so that they were aligned in the design. Empty constructs were subjected to a force that caused up to a 10% change in the original dimensions of structure. For each layer thickness, 12 constructs of each size were tested in both vertical and horizontal orientations. The compression tests of the constructs were carried out on a computer-controlled Zwick/Roell Z005 dynamometer (Barcelona, Spain), equipped with a load cell capacity of 5 KN (equivalent to 500 kg-force or kiloponds (Kp)). The test was conducted with a crosshead displacement speed of 0.005 mm/s [36]. The test conditions were maintained at room temperature (25 °C) and 50% humidity. The dynamometer was used to measure the load applied at breaking point (kg) and the compression (mm) before breaking. The breaking point was established with a force change threshold of 5%, a preload of 0.01 MPa, a compression speed of 1 mm/min, a test speed of 10 mm/min, and a maximum deformation in compression of 10% of its original dimensions.

The surface morphology of the 3D-printed model constructs was analyzed using an optical microscope (Leica EZ4 HD, Wetzlar, Germany) and scanning electron microscopy (SEM). Briefly, the samples were mounted on double carbon adhesive tape, securely fixed to brass holders, and coated with a thin layer of platinum. Imaging was conducted using a HITACHI S-4800 scanning electron microscope (Tokyo, Japan) equipped with a field emission gun (FEG) and a Bruke RX detector (Billerica, MA, USA) at an accelerating voltage of 5 KV. The imaging resolution achieved was 1.4 nm at 1 KV.

### 2.4. In Vitro Release Studies

To investigate the influence of layer thickness on antibacterial release, firstly in vitro release studies were carried out using 1.20 cm^3^ cylinders.

The in vitro release studies were performed using a dissolution testing apparatus (Erweka DT-80 Series, Langen, Germany). The 3D-printed constructs were loaded with 250 µL of a 1 mg/mL solution of MB used as a model drug. The choice of MB as a drug model was driven by its well-established utility as a model compound for the preliminary analysis of release profiles via spectrophotometric methods [36,39]. This approach allows for the rapid, reliable, and reproducible quantification of release kinetics, providing a foundational basis for subsequent, more targeted investigations [40,41,42]. As the 3D structure printed is enclosed, when the printing process reached 97% completion of the construct, the printing was paused and the cylinder was filled with 250 µL of a 1 mg/mL PBS (pH 7.4) solution of MB. After introducing the desired volume into the construct, the printing process was resumed, thereby retaining the solution inside the model for the temporary prosthesis. This ensures that once printing is complete, the solution is contained within the structure.

The equipment vessels were filled with 100 mL of phosphate buffer solution (PBS) at pH 7.4 and maintained at 37 °C (body temperature) with constant movement of the fluid (agitation rate of 75 rpm). It was carried out in this low volume to ensure detection of MB in the spectrophotometer, as large volumes can dilute the MB so much that it is not detected during the first moments of release. Samples of 1 mL were taken at predetermined intervals of 0.5, 1, 2, 4, 6, 24, 30, 48, and 54 h. Following each sample collection, 1 mL of fresh PBS solution was added to the corresponding vessel to maintain a constant volume. The amount of MB in the samples was quantified by UV spectrophotometry (Thermo Fisher Scientific GENESYS 20S, Waltham, MA, USA) at 660 nm. Calibration curves were obtained at concentrations of 0.01, 0.05, 0.1, 0.5, 1, 5, and 10 µg/mL. The cumulative amounts were determined by adding up the measured values to establish the MB release curve. Subsequently, the percentage of MB release was calculated.

Different mathematical models were employed to study drug release from the 3D-printed models. The zero-order, first-order, Higuchi, and Korsmeyer–Peppas models were evaluated to fit the experimental data. These equations are commonly used for drug release kinetic modeling [43,44].

### 2.5. Microbiological Studies

Microbiological studies were performed to evaluate the inhibition effect of antibacterials (CLOX and VAN) released from spacers printed with different layer thicknesses. Upon reaching 97% completion of the printing process, a brief pause allowed for the introduction of 250 µL of a 1 mg/mL antibacterial solution (in PBS, pH 7.4) into the cylindrical constructs. After the desired volume was added, the printing process was resumed, effectively encapsulating the solution within the model intended for use as a temporal prothesis.

Constructs, both with and without antibacterials, were immersed in a liquid nutrient broth (Mueller–Hinton), which contained a 1/10 solution of *Staphylococcus aureus* CET 239 at 0.5 McFarland (1.50 × 10^8^ CFU/mL). The 0.5 McFarland solution was prepared using a nephelometer (CristalSpecTM, Becton-Dickinson, Franklin Lakes, NJ, USA) and diluted by adding 4.5 mL of Mueller–Hinton medium to 0.5 mL. The studies included a total of 6 constructs for each layer thickness. The experiments were conducted for each antibacterial (CLOX and VAN) tested separately. The Mueller–Hinton broth was prepared according to ISO standards (Scharlab, Sentmenat, Barcelona, Spain). The samples were incubated for 24 h at 37 °C. To quantify the bacterial proliferation, the broth was diluted 1/100 and spread onto an agar plate. The plate was then incubated overnight at 37 °C to facilitate the growth and counting of colony-forming units (CFUs) [45,46]. Each experiment had two control solutions, both inoculated with a 1/10 solution of *S. aureus* at 0.5 McFarland: a negative control containing 250 µL of antibacterial solution (1 mg/mL) and a positive control with no antibacterials added. Bacterial growth was determined using turbidimetry, measured with a UV spectrophotometer (Thermo Fisher Scientific GENESYS 20S) at a wavelength of 600 nm [47].

### 2.6. Statistical Analysis

Microbiological data were expressed as mean ± standard variation. Statistical analysis was carried out by means of the Kruskal–Wallis tests with post hoc comparisons using the Mann–Whitney test with Bonferroni’s correction. In all case, the significance level was *p* < 0.05.

## 3. Results and Discussion 

Some authors have extensively examined the influence of layer thickness in 3D printing. Meiabadi et al. found that a layer thickness of 0.28 mm was the optimal setting to enhance the reproducibility of PLA printed parts [48]. Another study using different printing materials concluded that a layer thickness of 0.3 mm was optimal for their printed belts [49]. However, other important considerations in 3D printing include material selection, infill density, and print orientation [50]. These parameters were previously studied in order to select a 3D-printed construct with adequate characteristics [36]. In this study, three separate investigations were conducted: physical characterization, in vitro release studies, and microbiological studies. These studies examined three different print layer thicknesses: 0.2, 0.3 and 0.4 mm.

### 3.1. Physical Characterizations

The 3D-printed constructs were subjected to vertical and horizontal testing to evaluate their load-bearing capacity. The force was applied using the Zwick/Roell Z005 dynamometer. When the properties of the 3D-printed constructs were tested in a horizontal orientation, differences in strength were observed. This can be attributed to the alignment of the force vectors. Specifically, in vertical force applications, resistance is enhanced as the forces are distributed over the base of the cylinder. Additionally, it has been noted that layers printed horizontally throughout the cylindrical structure exhibit a tendency to separate more readily under force compared to those aligned vertically.

Table 2 presents the mean breaking load and compression values for the two sizes of the 3D constructs examined, at the various layer thicknesses tested, in both horizontal and vertical orientations.

**Figure 2:**
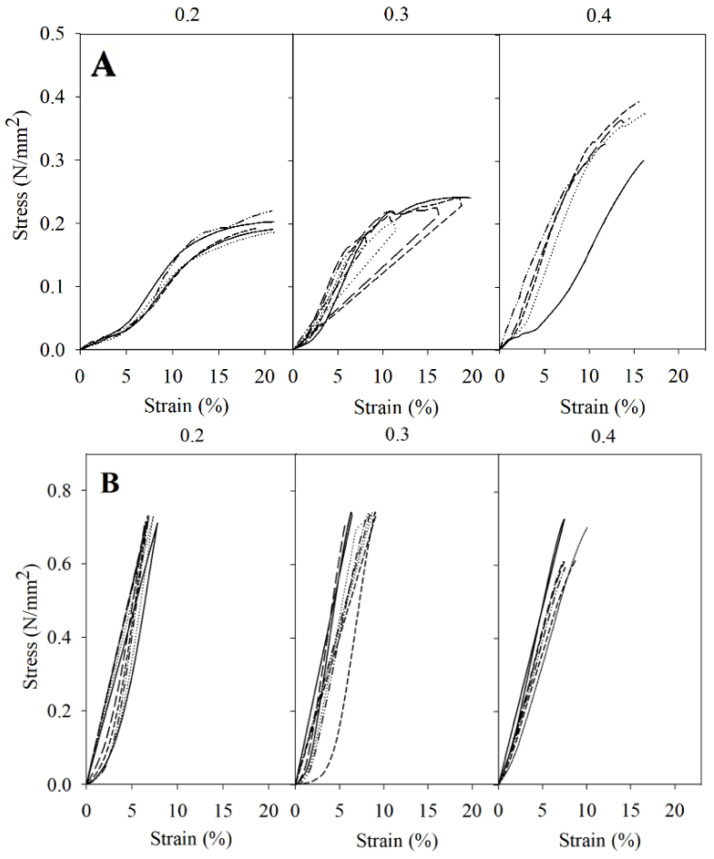
Representation of stress–strain for the 23.12 cm^3^ spacers in horizontal (**A**) and vertical (**B**) position for each layer thickness (*n* = 6) using the Zwick/Roell Z005 dynamometer.

**Figure 3:**
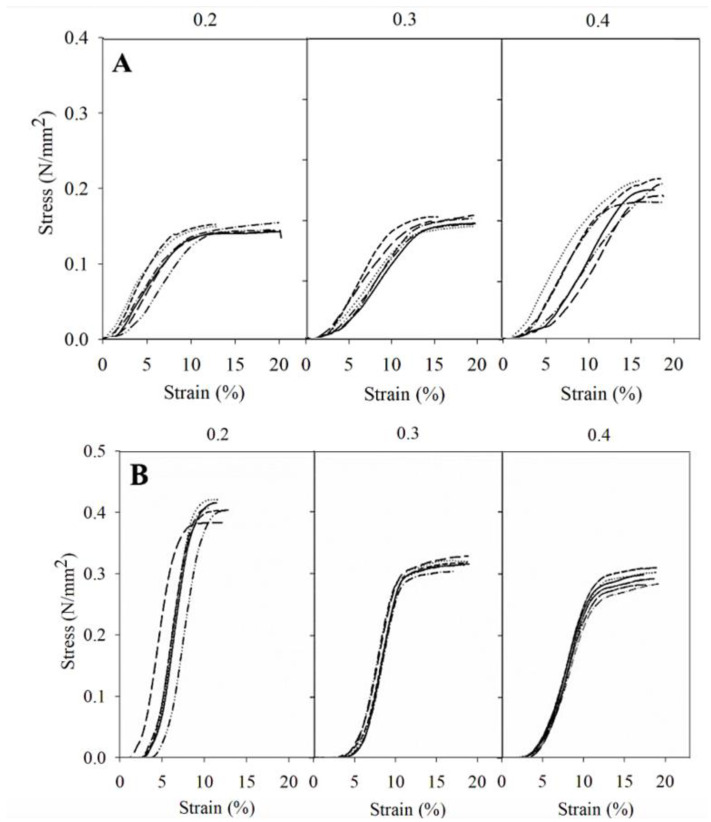
Representation of stress–strain for the 1.20 cm^3^ spacers in horizontal (**A**) and vertical (**B**) position for each layer thickness (*n* = 6) using the Zwick/Roell Z005 dynamometer.

For components experiencing tension or compression, understanding the mechanical properties of stress and strain is crucial for assessing their ability to withstand applied loads. Stress represents the force exerted on a material per unit area, while strain measures the resulting deformation or displacement caused by this stress. The stress–strain representation serves as a critical tool, offering insights into the material’s capacity to resist external forces before undergoing permanent deformation or failure. The stress-strain representations for the two PLA cylinders tested, as shown in Figure 2 and Figure 3, exhibit noticeable differences. This emphasizes the impact of the cylinders’ volume size and layer height on the material’s ability to withstand external forces prior to undergoing permanent deformation or fracture.

The test was conducted with two force limits: one was automatically stopped when the resistance of the force exerted decreased by 5% and the other at 15% (was deformed or broken) (*n* = 6). Both figures show different results between the two constructs, which only differ in their volume, the large one being more resistant to compression. These differences may be explained by the number of layers. Reducing the size of a printed product derived from a larger one results in a reduced number of layers, as the filament ratio remains consistent regardless of the chosen layer thickness. Thus, a smaller structure results in lower strength, as it has to rely upon fewer layers for its reinforcement [34].

In the 23.12 cm^3^ constructs, the horizontal tests showed that the constructs with a layer thickness of 0.4 mm required greater force to rupture. In the case of the vertical test, all cylinders were able to sustain the maximum force (500 Kp or 0.734 N/mm^2^) that the dynamometer could apply in the experiment without breaking. In the case of the 1.20 cm^3^ constructs, the results also showed that horizontally, the 0.4 mm constructs were more resistant to breakage. In the vertical test, the cylinders with a layer thickness of 0.2 mm were the most resistant. Figure 3 reveals that when the force is applied horizontally, the thicker the layer, the lower the force required for deformation, and therefore less resistant. These findings are particularly relevant for the design of prosthetic spacers, as they provide insights into the load-bearing capacity of 3D-printed constructs at different layer thicknesses, mimicking real-world scenarios.

In summary, by increasing the layer thickness horizontally in both sizes, the force is better distributed, with constructions of a layer thickness of 0.4 mm being the most resistant. This finding aligns with previous studies that have explored the impact of layer thickness on the strength of PLA printed materials, suggesting that adjustments in layer thickness can significantly influence the strength of the material [51,52]. However, our results also indicate complex behavior depending on the orientation and volume of the constructs. For the larger, 23.12 cm^3^ constructs, the variation in layer thickness does not appear to impact resistance significantly, whereas the smaller 1.20 cm^3^ constructions exhibit maximum resistance at a layer thickness of 0.2 mm. These observations contribute to the current understanding by providing insight into the complexity of how layer thickness affects the mechanical properties of 3D-printed materials. Unlike the uniform trend suggested by prior research, our findings underscore the importance of considering the dimensions and orientation of the printed object. This is particularly relevant in the context of another study that developed rock-like specimens using sand powder 3D printing, which found that an increase in layer thickness led to interlayer overlapping, adversely affecting properties such as weight, density, and uniaxial compressive strength [53]. The reduction in peak strength and elastic modulus observed at layer thicknesses of 0.3 and 0.4 mm, compared to 0.2 mm, highlights the potential resistance involved in selecting layer thickness for optimizing the physical and mechanical properties of 3D-printed constructs. Furthermore, Kuznetsov and colleagues identified layer thickness as a critical factor influencing intralayer cohesion, with resistance decreasing as layer thickness increases [54]. This complements our findings and helps to elucidate, at least partially, the underlying mechanisms that may contribute to the observed differences in material resistance based on layer thickness and orientation. In short, while thicker layers might provide certain structural benefits, they could also compromise material cohesion and, by extension, mechanical integrity.

**Figure 4:**
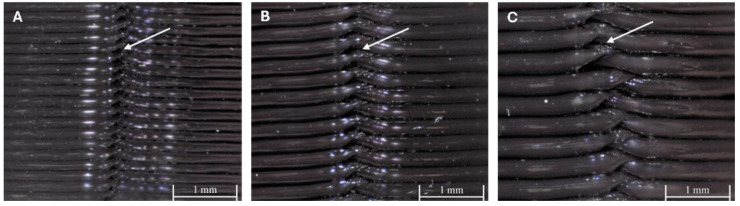
Optical microscope images of the (**A**) 0.2, (**B**) 0.3 and (**C**) 0.4 mm layer thickness at 25× magnification.

**Figure 5:**
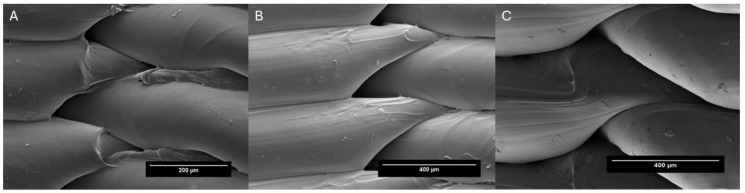
Electron microscope images of (**A**) 0.2 mm layer at 150× magnification and (**B**) 0.3 and (**C**) 0.4 mm layer at 300× magnification to show how each layer of filament superimposes over the next to create pores.

Figure 4 shows layers of 1.20 cm^3^ cylinders with different layer thicknesses viewed with optical microscopy. Figure 5 (SEM) shows how the layers of 23.12 mm cylinders superimpose with a layer thickness of 0.3 mm. With the layer thickness, printing speed, and number of layers, the porosity surface could be modified, allowing the control of drug release to achieve its therapeutic target. SEM allows visualization of the superimposition of the printed layers as well as the pores.

From the SEM images, it can be seen that the PLA building layers are well surface-bonded. The regions where layers intersect function as porous conduits. The intricate arrangement of the lines creates a network of interconnected channels that allows for the controlled and gradual release of the introduced substances. Previous research studies have demonstrated the effective incorporation of silver nanoparticles, contraceptive drugs, and chitosan/sodium alginate into 3D-printed structures, each exhibiting unique morphological characteristics and drug dispersion behavior upon analysis in SEM microscopic studies [55,56,57]. In summary, SEM microscopy has allowed us to confirm that pores are formed at the points at which the filament layers superimpose one another.

Using SEM, a detailed analysis of the pores present in the structures as well as pore measurements were performed. The pore size calculated for each cylinder was 390.00 ± 20.60 µm^2^ for the 0.2 mm layer height, 1540.50 ±14.11 µm^2^ for the 0.3 mm layer height, and 2691.50 ± 12.98 µm^2^ for the 0.4 mm layer height. When multiplied by the total number of layers, this resulted in 23.40 mm^2^, 61,62 mm^2^, and 80.74 mm^2^ for 0.2 mm, 0.3 mm, and 0.4 mm, respectively. This represents 3.43%, 9.05%, and 11.80% of the total cylinder surface area, respectively. All these calculations were made according to Bueno-López (2021) [36].

These results correlate with the increase in release as the layer height increases with the antimicrobial effect and provide valuable information on the morphology and potential functionality of 3D-printed constructs in relation to their ability to release antibiotics.

### 3.2. Release Studies

An evaluation of the in vitro release of substances was carried out to assess the release capacity of the constructs upon contact with an aqueous medium. The amount of methylene blue (MB) released was calculated from a calibration curve with concentrations ranging from 0.01 to 10 µg/mL of MB. The release profiles of the 3D constructs are presented in Figure 5.

**Figure 6:**
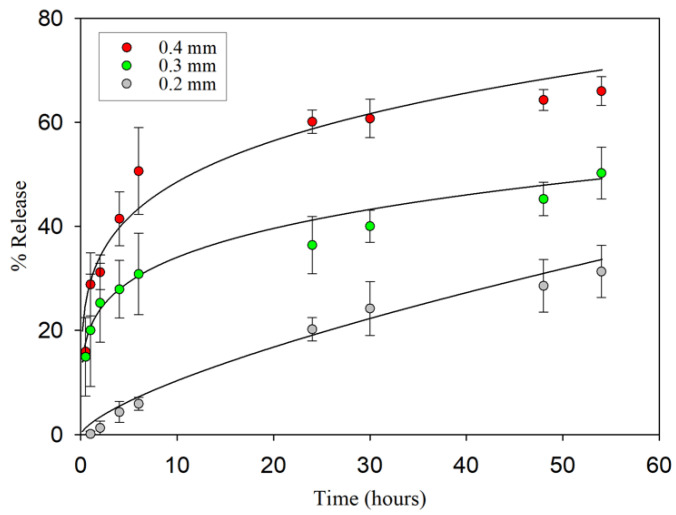
Percentage of MB released from the 1.20 cm^3^ 3D constructs printed with a layer thickness of 0.2 mm, 0.3 mm, and 0.4 mm for up to 54 h. The regression lines are fitted using the Korsmeyer–Peppas model.

The results are expressed as the cumulative percentage of MB released at each time point analyzed (*n* = 3, mean ± sd, Figure 6). The release kinetics were examined using several mathematical models, with the Korsmeyer–Peppas model being identified as the most suitable equation to characterize MB release from 3D constructs with different layer thicknesses. Figure 6 displays the percentage of MB released according to this mathematical model. The MB release during the first two hours reached 1.260 ± 0.327% when the layer thickness was 0.2 mm, 25.225 ± 1.827% for a thickness of 0.3 mm, and 31.193 ± 3.338% when the layer thickness was 0.4 mm. After 54 h, the highest percentage of release obtained with the 0.2 mm thickness was 31.333 ± 4.997%, while 50.258 ± 4.938% and 66.013 ± 2.760% were released from the 0.3- and 0.4-layer thickness, respectively. The observed release profiles have direct implications for the development of drug-eluting prosthetic spacers, where controlling drug release is crucial for therapeutic efficacy. As can be seen in Figure 6, the amount of MB was released differently depending on the layer thickness. Our results are in agreement with several release studies using 3D printing models that demonstrate that this type of construct provides sustained release over time [58,59,60].

It is crucial to acknowledge that while our experimental setup does not directly simulate clinical conditions—given that the constructs were immersed in a liquid medium rather than interacting with the complex biological environment surrounding a temporary prosthesis—the methodology employed effectively demonstrates the capacity for controlled drug release from the pores of the cylinder. Other aspects such as building patterns are critical in determining the physical and mechanical properties of the printed structures, as well as their functional performance in terms of pore characteristics of the structure and the release profiles [61,62,63]. However, our research establishes that layer thickness is a decisive factor in shaping the release profile, a principle that is in line with the results of previous studies investigating the influence of layer thickness in PLA 3D-printed structures [64,65,66,67,68]. Yang et al. developed a 3D-printed implant using PLA with a cytotoxic agent inside and concluded that the thicker the layer, the greater the release, being 86–97% in the first 2 h [64]. Other authors conducted a release study using multicompartmental PVA capsules for oral administration and obtained different results (78–81%) depending on this impression parameter [65]. Others tested the release profile of 0.3, 0.2, and 0.1 mm printed tablets and reported that an increase in the layer thickness resulted in a faster dissolution rate, being released over 65–96%, depending on layer thickness after 8 h of release [66]. Additional research found a high percentage of drug release at 10 min by increasing the layer thickness from 80 to 120 µm in solid 3D-printed oral forms [67]. In summary, increasing the layer thickness provides a higher level of porosity.

### 3.3. Microbiological Studies

In our study, microbiological tests were carried out to evaluate whether antibacterials incorporated into 3D constructs were released and were able to inhibit bacterial growth.

**Figure 7:**
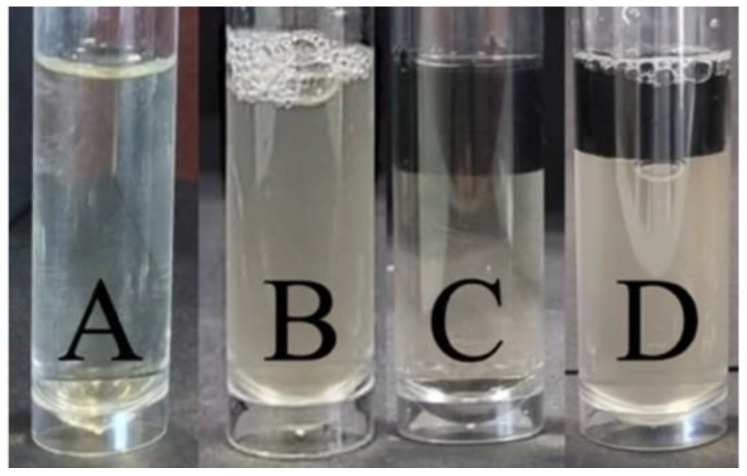
0.5 McFarland standard 1/10 dilution of *Staphylococcus aureus* test tubes after 24 h of incubation. (**A**) negative control (*S. aureus* and CLOX in solution); (**B**) positive control (*S. aureus*, no antibacterial); (**C**) 3D constructs loaded with CLOX; (**D**) 3D constructs without CLOX. The 3D constructs in this image have been printed with a layer thickness of 0.2 mm.

Figure 7 shows the turbidity of the negative (Figure 7A) and positive (Figure 7B) control samples compared to those containing the 3D constructs with CLOX (Figure 7C) and without the antibacterial (Figure 7D). The turbidity of the sample with the constructs containing cloxacillin (Figure 7C) is similar to that of the negative controls (Figure 7A). This situation occurs in all the 3D constructs printed with the three different layer thicknesses tested and with both CLOX and VAN. This indicates that the antibacterials present in the cylinders (CLOX and VAN) have permeated through and inhibited bacterial growth.

**Figure 8:**
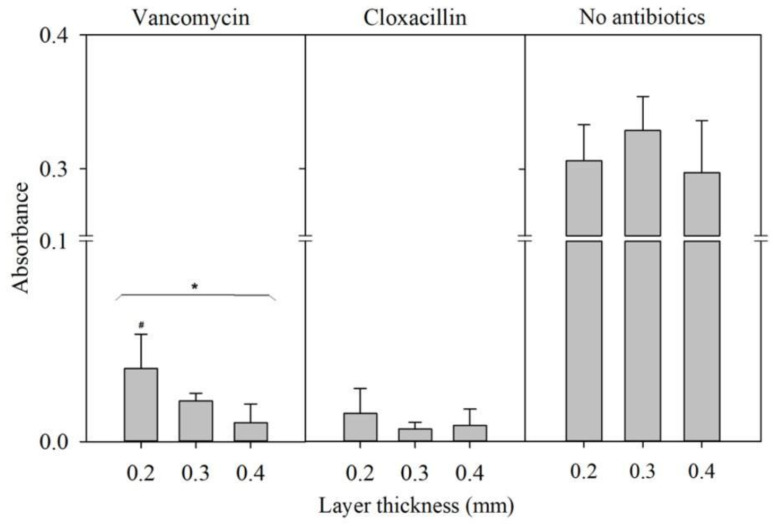
Absorbance for each 3D construct printed with different layer thicknesses (0.2, 0.3, and 0.4 mm) loaded with the antibacterials VAN and CLOX and constructs without loaded antibacterials (no antibacterials) after being incubated for 24 h to allow bacterial growth. * Indicates the statistical differences between the three layer thicknesses (*p* = 0.01); # indicates the differences between 0.2 and 0.4 mm (*p* < 0.05).

The bacterial presence was determined using turbidimetry at a wavelength of 600 nm [41]. The results are illustrated in Figure 8, which shows the mean absorbance for each 3D construct printed with different layer thicknesses (0.2, 0.3, and 0.4 mm) with VAN and CLOX and without antibacterials (*n* = 6).

Samples containing antibacterials displayed minimal absorbance, implying that the antibacterials had diffused from the constructs, thereby inhibiting bacterial growth and a decrease in turbidity. In contrast, samples with no antibacterials displayed absorbance values of approximately 0.3. No statistical differences were observed among the CLOX constructs. Significant differences were observed in the VAN experiments comparing the 3D constructs printed with different layer thicknesses (*p* = 0.01; Kruskal–Wallis test). However, post hoc tests (Mann–Whitney test applying Bonferroni’s correction) found significant differences between 0.4 and 0.2 mm, but failed to detect significant differences between layer thicknesses of 0.2 and 0.3 mm. These findings underscore the potential of 3D-printed prosthetic spacers loaded with antibacterials to effectively inhibit bacterial growth, a critical aspect in preventing post-operative infections in joint replacement surgeries.

VAN and CLOX are structurally distinct antibacterials. VAN is a glycopeptide and has a complex structure characterized by a large cyclic heptapeptide core with various sugar residues. CLOX, on the other hand, belongs to the penicillin class of antibacterials, featuring a β-lactam ring and a side chain. The structure of methylene blue is a heterocyclic aromatic ring system with a central nitrogen atom. Therefore, VAN has a far more complex structure than CLOX and MB. As such, the molar mass for CLOX and MB is 435.882 g/mol and 319.85 g/mol respectively, whereas VAN has a molar mas of 1449.3 g/mol. The different microbiological results of the antibacterials show a faster release of CLOX, resulting in a decrease in turbidity compared to that of VAN. This may be due to the difference in the structural complexity of VAN compared to CLOX.

Positive control samples, containing bacteria without any antibacterials, displayed massive growth, underscoring the efficacy of the antibacterial agents when incorporated into the 3D constructs. These findings were further supported by the absence of bacterial colonies in samples with antibacterials, highlighting the potential of 3D-printed constructs as carriers for sustained antibacterial delivery. This research aligns with previous studies demonstrating the utility of 3D printing technology in creating drug delivery systems and medical devices with antibacterial agents incorporated. Aldrich and co-workers conducted microbiological trials with polycaprolactone scaffolds infused with rifampicin and daptomycin for the treatment of infections in craniotomies. Results in mice studies revealed a reduction in colony-forming units (CFUs) in samples treated with the antibacterial-infused design compared to systemic treatment [69]. Other authors used a 3D-printed patch design with three different antibacterials (amoxicillin, kanamycin, and ampicillin). They achieved sustained antibacterial release for up to 336 h and observed inhibition halos for *S. aureus* and *E. coli*, demonstrating the antimicrobial activity of these antibacterial-incorporated designs [52]. In another study, 3D-printed dental materials were tested for antimicrobial activity with and without antibacterial incorporation, and statistically significant differences in the number of bacterial colonies (CFU) were observed between these two groups [70]. The differential release patterns observed in our study underscore the importance of considering the physical and chemical properties of both antibacterial agents and printing materials to optimize therapeutic outcomes.

## 4. Conclusions

This study evaluated the effects of different layer thicknesses in a defined spacer 3D construct (0.2, 0.3, and 0.4 mm). Physical characterization studies showed that increasing layer thickness results in a higher force required to reach the breaking point in a horizontal position, with constructions of a layer thickness of 0.4 mm being the most resistant. Different results were obtained depending on the construct dimensions when the force was applied in the vertical position. The PLA 3D-printed constructs were found to have sufficient strength to withstand a force of 500 kg-force, which is more than they would be subjected to if they were to be used in vivo as prosthesis. The in vitro release of MB, used as a model drug, varied depending on layer thickness. Specifically, thicker layers result in greater quantities of drugs being released from the constructs used as model spacers, which is related to an increase in porosity. In terms of the effects of antibacterials incorporated into the model spacers, CLOX and VAN were released from the 3D constructs and inhibited bacterial growth effectively. These findings demonstrate that adjusting layer thickness, a 3D printing parameter, enables the generation of varying profiles for antimicrobial release, affecting its efficacy. Overall, these findings show the significance of optimizing layer thickness in 3D-printed prosthetic spacers to achieve good mechanical properties, controlled drug release, and effective antimicrobial potency.

## Figures and Tables

**Table 1:** Principal printing parameters of Flashforge Creator Pro printer and cylinder dimensions.

Printing temperature	200 °C
Platform temperature	60 °C
Print speed	60 mm/s
Layer thickness	0.2, 0.3, and 0.4 mm
Infill	10%
Small cylinder	Height: 11.00 mm Diameter: 11.80 mm External wall thickness: 1.35 mm Total volume: 1.20 cm^3^ Capacity up to fill: 268 µL
Large cylinder	Height: 30.14 mm Diameter: 31.26 mm External wall thickness: 1.37 mm Total volume: 23.12 cm^3^ Total capacity up to fill: 16.5 mL

**Table 2:** Values of breaking load and compression of the two sizes of the 3D constructs (1.20 and 23.12 cm^3^) at different layer thicknesses (0.2, 0.3, and 0.4 mm) in horizontal and vertical orientation.

Construct Size	Orientation Test	Layer Thickness (mm)	Breaking Load (Mean ± sd) (Kp)	Compression (Mean ± SD) (mm)
23.12 cm^3^	Horizontal	0.2	137.1 ± 5.9	3.6 ± 0.9
		0.3	140.0 ± 17.7	1.8 ± 0.6
		0.4	244.4 ± 23.2	3.2 ±0.9
	Vertical	0.2	490.6 ± 7.4	0.8 ± 0.1
		0.3	500.3 ± 2.3	0.9 ± 0.2
		0.4	472.5 ± 13.9	1.2 ± 0.1
1.20 cm^3^	Horizontal	0.2	92.6 ± 5.1	1.5 ± 0.3
		0.3	99.0 ± 3.8	1.6 ± 0.1
		0.4	125.0 ± 7.7	1.6 ± 0.1
	Vertical	0.2	202.5 ± 6.3	1.0 ± 0.1
		0.3	158.1 ± 4.1	1.5 ± 0.1
		0.4	147.4 ± 5.4	1.5 ± 0.1

## Data Availability

The original contributions presented in the study are included in the article, further inquiries can be directed to the corresponding author.

## References

[1] Mcconoughey S.J., Howlin R., Granger J.F., Manring M.M., Calhoun J.H., Shirtlif M., Kathju S., Stoodley P. (2014). Biofilms in Periprosthetic Orthopedic Infections HHS Public Access. Future Microbiol..

[2] Kunutsor S.K., Whitehouse M.R., Blom A.W., Beswick A.D. (2016). Patient-Related Risk Factors for Periprosthetic Joint Infection after Total Joint Arthroplasty: A Systematic Review and Meta-Analysis. PLoS ONE.

[3] Roy R., Tiwari M., Donelli G., Tiwari V. (2018). Strategies for Combating Bacterial Biofilms: A Focus on Anti-Biofilm Agents and Their Mechanisms of Action. Virulence.

[4] Flemming H.C., Wingender J. (2010). The Biofilm Matrix. Nat. Rev. Microbiol..

[5] Tsai Y., Chang C.H., Lin Y.C., Lee S.H., Hsieh P.H., Chang Y. (2019). Different Microbiological Profiles between Hip and Knee Prosthetic Joint Infections. J. Orthop. Surg..

[6] Papadimitriou-Olivgeris M., Senn L., Bertelli C., Grandbastien B., Steinmetz S., Boillat-Blanco N. (2022). Prevalence and Factors Associated with Prosthetic Joint Infections in Patients with *Staphylococcus aureus* Bacteraemia: A 7-Year Retrospective Study. Antibiotics.

[7] Zardi E.M., Franceschi F. (2020). Prosthetic Joint Infection. A Relevant Public Health Issue. J. Infect. Public Health.

[8] Tözün I.R., Ozden V.E., Dikmen G., Karaytuğ K. (2020). Trends in the Treatment of Infected Knee Arthroplasty. EFORT Open Rev..

[9] Basile G., Gallina M., Passeri A., Gaudio R.M., Castelnuovo N., Ferrante P., Calori G.M. (2021). Prosthetic Joint Infections and Legal Disputes: A Threat to the Future of Prosthetic Orthopedics. J. Orthop. Traumatol..

[10] Dunne N.J., Hill J., McAfee P., Kirkpatrick R., Patrick S., Tunney M. (2008). Incorporation of Large Amounts of Gentamicin Sulphate into Acrylic Bone Cement: Effect on Handling and Mechanical Properties, Antibiotic Release, and Biofilm Formation. Proc. Inst. Mech. Eng. Part H J. Eng. Med..

[11] Berberich C., Josse J., Ruiz P.S. (2022). Patients at a High Risk of PJI: Can We Reduce the Incidence of Infection Using Dual Antibiotic-Loaded Bone Cement?. Arthroplasty.

[12] Wang J., Zhu C., Cheng T., Peng X., Zhang W., Qin H., Zhang X. (2013). A Systematic Review and Meta-Analysis of Antibiotic-Impregnated Bone Cement Use in Primary Total Hip or Knee Arthroplasty. PLoS ONE.

[13] Xie H., Liu Y., An H., Yi J., Li C., Wang X., Chai W. (2022). Recent Advances in Prevention, Detection and Treatment in Prosthetic Joint Infections of Bioactive Materials. Front. Bioeng. Biotechnol..

[14] Mazzucchelli L., Rosso F., Marmotti A., Bonasia D.E., Bruzzone M., Rossi R. (2015). The Use of Spacers (Static and Mobile) in Infection Knee Arthroplasty. Curr. Rev. Musculoskelet. Med..

[15] Samelis P.V., Papagrigorakis E., Sameli E., Mavrogenis A., Savvidou O., Koulouvaris P. (2022). Current Concepts on the Application, Pharmacokinetics and Complications of Antibiotic-Loaded Cement Spacers in the Treatment of Prosthetic Joint Infections. Cureus.

[16] Kilinç S., Tunç T., Pazarci Ö., Sümer Z. (2020). Research into Biocompatibility and Cytotoxicity of Daptomycin, Gentamicin, Vancomycin and Teicoplanin Antibiotics at Common Doses Added to Bone Cement. Jt. Dis. Relat. Surg..

[17] Cara A., Ballet M., Hemery C., Ferry T., Laurent F., Josse J. (2021). Antibiotics in Bone Cements Used for Prosthesis Fixation: An Efficient Way to Prevent *Staphylococcus aureus* and *Staphylococcus epidermidis* Prosthetic Joint Infection. Front. Med..

[18] Schmitt D.R., Killen C., Murphy M., Perry M., Romano J., Brown N. (2020). The Impact of Antibiotic-Loaded Bone Cement on Antibiotic Resistance in Periprosthetic Knee Infections. CiOS Clin. Orthop. Surg..

[19] Holleyman R.J., Deehan D.J., Walker L., Charlett A., Samuel J., Shirley M.D.F., Baker P.N. (2019). Staphylococcal Resistance Profiles in Deep Infection Following Primary Hip and Knee Arthroplasty: A Study Using the NJR Dataset. Arch. Orthop. Trauma. Surg..

[20] Mensah L.M., Love B.J. (2021). A Meta-Analysis of Bone Cement Mediated Antibiotic Release: Overkill, but a Viable Approach to Eradicate Osteomyelitis and Other Infections Tied to Open Procedures. Mater. Sci. Eng. C.

[21] Tan D.K., Maniruzzaman M., Nokhodchi A. (2020). Development and Optimisation of Novel Polymeric Compositions for Sustained Release Theophylline. Polymers.

[22] Jamróz W., Szafraniec J., Kurek M., Jachowicz R. (2018). 3D Printing in Pharmaceutical and Medical Applications. Pharm. Res..

[23] Attaeyan A., Shahgholi M., Khandan A. (2024). Fabrication and Characterization of Novel 3D Porous Titanium-6Al-4V Scaffold for Orthopedic Application Using Selective Laser Melting Technique. Iran. J. Chem. Chem. Eng..

[24] Zhu X., Li H., Huang L., Zhang M., Fan W., Cui L. (2020). 3D Printing Promotes the Development of Drugs. Biomed. Pharmacother..

[25] Beslikas T., Gigis I., Goulios V., Christoforides J., Papageorgiou G.Z., Bikiaris D.N. (2011). Crystallization Study and Comparative in Vitro–in Vivo Hydrolysis of PLA Reinforcement Ligament. Int. J. Mol. Sci..

[26] Weisman J.A., Nicholson J.C., Tappa K., Jammalamadaka U., Wilson C.G., Mills D.K. (2015). Antibiotic and Chemotherapeutic Enhanced Three-Dimensional Printer Filaments and Constructs for Biomedical Applications. Int. J. Nanomed..

[27] Benmassaoud M.M., Kohama C., Kim T.W.B., Kadlowec J.A., Foltiny B., Mercurio T., Ranganathan S.I. (2019). Efficacy of Eluted Antibiotics through 3D Printed Femoral Implants. Biomed. Microdevices.

[28] Amekyeh H., Tarlochan F., Billa N. (2021). Practicality of 3D Printed Personalized Medicines in Therapeutics. Front. Pharmacol..

[29] Elkasabgy N.A., Mahmoud A.A., Maged A. (2020). 3D Printing: An Appealing Route for Customized Drug Delivery Systems. Int. J. Pharm..

[30] Vaz V.M., Kumar L. (2021). 3D Printing as a Promising Tool in Personalized Medicine. AAPS PharmSciTech.

[31] Muhammad A.R., Sakura R.R., Dwilaksana D., Trifiananto M. (2022). Layer Height, Temperature Nozzle, Infill Geometry and Printing Speed Effect on Accuracy 3D Printing PETG. REM J..

[32] Nguyen Q.B., Luu D.N., Nai S.M.L., Zhu Z., Chen Z., Wei J. (2018). The Role of Powder Layer Thickness on the Quality of SLM Printed Parts. Arch. Civ. Mech. Eng..

[33] Farzadi A., Solati-Hashjin M., Asadi-Eydivand M., Osman N.A.A. (2014). Effect of Layer Thickness and Printing Orientation on Mechanical Properties and Dimensional Accuracy of 3D Printed Porous Samples for Bone Tissue Engineering. PLoS ONE.

[34] Zhang Z.C., Li P.l., Chu F.t., Shen G. (2019). Influence of the Three-Dimensional Printing Technique and Printing Layer Thickness on Model Accuracy. J. Orofac. Orthop..

[35] Elkaseer A., Schneider S., Scholz S.G. (2020). Experiment-Based Process Modeling and Optimization for High-Quality and Resource-Efficient FFF 3D Printing. Appl. Sci..

[36] Bueno-López C., Tamarit-Martínez C., Alambiaga-Caravaca A.M., Balaguer-Fernández C., Merino V., López-Castellano A., Rodilla V. (2021). 3D Printing of Temporary Prostheses for Controlled-Release of Drugs: Design, Physical Characterization and Preliminary Studies. Pharmaceuticals.

[37] Bembenek M., Kowalski Ł., Kosoń-Schab A. (2022). Research on the Influence of Processing Parameters on the Specific Tensile Strength of FDM Additive Manufactured PET-G and PLA Materials. Polymers.

[38] Hsueh M.H., Lai C.J., Wang S.H., Zeng Y.S., Hsieh C.H., Pan C.Y., Huang W.C. (2021). Effect of Printing Parameters on the Thermal and Mechanical Properties of 3d-Printed Pla and Petg, Using Fused Deposition Modeling. Polymers.

[39] El-Ries M.A., Khaled E., Zidane F.I., Ibrahim S.A., Abd-Elmonem M.S. (2012). Catalytic Spectrophotometric Determination of Iodide in Pharmaceutical Preparations and Edible Salt. Drug Test. Anal..

[40] Li D., Wang M., Song W.L., Yu D.G., Bligh S.W.A. (2021). Electrospun Janus Beads-on-a-String Structures for Different Types of Controlled Release Profiles of Double Drugs. Biomolecules.

[41] Zhong Y., Whittington C.F., Zhang L., Haynie D.T. (2007). Controlled Loading and Release of a Model Drug from Polypeptide Multilayer Nanofilms. Nanomedicine.

[42] Jiang B., Li B., Li Biomaterials B. (2009). Tunable Drug Loading and Release from Polypeptide Multilayer Nanofi Lms. Int. J. Nanomed..

[43] Siepmann J., Siepmann F. (2008). Mathematical Modeling of Drug Delivery. Int. J. Pharm..

[44] Fu Y., Kao W.J. (2010). Drug Release Kinetics and Transport Mechanisms of Non-Degradable and Degradable Polymeric Delivery Systems. Expert Opin. Drug Deliv..

[45] Cockerill F.R., Wikler M.A., Alder J., Dudley M.N., Eliopoulos G.M., Ferraro M.J., Hardy D.J., Hecht D.W., Hindler J.A., Patel J.B. (2012). Methods for Dilution Antimicrobial Susceptibility Tests for Bacteria That Grow Aerobically.

[46] Martínez-Moreno J., Merino V., Nácher A., Rodrigo J.L., Bonet Yuste B.B., Merino-Sanjuán M. (2017). Bioactivity of Ceftazidime and Fluconazole Included in Polymethyl Methacrylate Bone Cement for Use in Arthroplasty. J. Arthroplast..

[47] Xu J., Li Y., Wang H., Zhu M., Feng W., Liang G. (2021). Enhanced Antibacterial and Anti-Biofilm Activities of Antimicrobial Peptides Modified Silver Nanoparticles. Int. J. Nanomed..

[48] Meiabadi M.S., Moradi M., Karamimoghadam M., Ardabili S., Bodaghi M., Shokri M., Mosavi A.H. (2021). Modeling the Producibility of 3d Printing in Polylactic Acid Using Artificial Neural Networks and Fused Filament Fabrication. Polymers.

[49] Wu W., Geng P., Li G., Zhao D., Zhang H., Zhao J. (2015). Influence of Layer Thickness and Raster Angle on the Mechanical Properties of 3D-Printed PEEK and a Comparative Mechanical Study between PEEK and ABS. Materials.

[50] Syrlybayev D., Zharylkassyn B., Seisekulova A., Akhmetov M., Perveen A., Talamona D. (2021). Optimisation of Strength Properties of FDM Printed Parts—A Critical Review. Polymers.

[51] Benli M., Eker-Gümüş B., Kahraman Y., Huck O., Özcan M. (2021). Can Polylactic Acid Be a CAD/CAM Material for Provisional Crown Restorations in Terms of Fit and Fracture Strength?. Dent. Mater. J..

[52] Vakharia V.S., Kuentz L., Salem A., Halbig M.C., Salem J.A., Singh M. (2021). Additive Manufacturing and Characterization of Metal Particulate Reinforced Polylactic Acid (Pla) Polymer Composites. Polymers.

[53] Xu Q., Jiang L., Ma C., Niu Q., Wang X. (2021). Effect of Layer Thickness on the Physical and Mechanical Properties of Sand Powder 3D Printing Specimens. Front. Earth Sci..

[54] Kuznetsov V.E., Solonin A.N., Urzhumtsev O.D., Schilling R., Tavitov A.G. (2018). Strength of PLA Components Fabricated with Fused Deposition Technology Using a Desktop 3D Printer as a Function of Geometrical Parameters of the Process. Polymers.

[55] Tzounis L., Bangeas P.I., Exadaktylos A., Petousis M. (2020). Three-Dimensional Printed Polylactic Acid (PLA) Surgical Retractors with Sonochemically Immobilized Silver Nanoparticles: The Next Generation of Low-Cost Antimicrobial Surgery Equipment. Nanomaterials.

[56] Long J., Nand A.V., Ray S., Mayhew S., White D., Bunt C.R., Seyfoddin A. (2018). Development of Customised 3D Printed Biodegradable Projectile for Administrating Extended-Release Contraceptive to Wildlife. Int. J. Pharm..

[57] Ilhan E., Ulag S., Sahin A., Karademir B., Ekren N., Kilic O., Sengor M., Kalaskar D.M., Nuzhet F., Gunduz O. (2021). Journal of the Mechanical Behavior of Biomedical Materials Fabrication of Tissue-Engineered Tympanic Membrane Patches Using 3D-Printing Technology. J. Mech. Behav. Biomed. Mater..

[58] Altun E., Yuca E., Ekren N., Kalaskar D.M., Ficai D., Dolete G., Ficai A., Gunduz O. (2021). Kinetic Release Studies of Antibiotic Patches for Local Transdermal Delivery. Pharmaceutics.

[59] Liaskoni A., Wildman R.D., Roberts C.J. (2021). 3D Printed Polymeric Drug-Eluting Implants. Int. J. Pharm..

[60] Chunate H.-T., Khamwannah J., Azeez A., Aliyu A., Tantavisut S., Puncreobutr C., Khamkongkaeo A., Tongyam C., Tumkhanon K., Phetrattanarangsi T. (2021). Titania Nanotube Architectures Synthesized on 3D-Printed Ti-6Al-4V Implant and Assessing Vancomycin Release Protocols. Materials.

[61] Patel S.K., Khoder M., Peak M., Alhnan M.A. (2021). Controlling Drug Release with Additive Manufacturing-Based Solutions. Adv. Drug Deliv. Rev..

[62] Prasad L.K., Smyth H. (2016). 3D Printing Technologies for Drug Delivery: A Review. Drug Dev. Ind. Pharm..

[63] Long J., Gholizadeh H., Lu J., Bunt C., Seyfoddin A. (2016). Application of Fused Deposition Modelling (FDM) Method of 3D Printing in Drug Delivery. Curr. Pharm. Des..

[64] Yang N., Chen H., Han H., Shen Y., Gu S., He Y., Guo S. (2018). 3D Printing and Coating to Fabricate a Hollow Bullet-Shaped Implant with Porous Surface for Controlled Cytoxan Release. Int. J. Pharm..

[65] Matijašić G., Gretić M., Vinčić J., Poropat A., Cuculić L., Rahelić T. (2019). Design and 3D Printing of Multi-Compartmental PVA Capsules for Drug Delivery. J. Drug Deliv. Sci. Technol..

[66] Sharma V., Shaik K.M., Choudhury A., Kumar P., Kala P., Sultana Y., Shukla R., Kumar D. (2021). Investigations of Process Parameters during Dissolution Studies of Drug Loaded 3D Printed Tablets. Proc. Inst. Mech. Eng. Part H J. Eng. Med..

[67] Gueche Y.A., Sanchez-Ballester N.M., Bataille B., Aubert A., Rossi J.C., Soulairol I. (2021). A Qbd Approach for Evaluating the Effect of Selective Laser Sintering Parameters on Printability and Properties of Solid Oral Forms. Pharmaceutics.

[68] Maver T., Mastnak T., Mihelič M., Maver U., Finšgar M. (2021). Clindamycin-Based 3D-Printed and Electrospun Coatings for Treatment of Implant-Related Infections. Materials.

[69] Aldrich A., Kuss M.A., Duan B., Kielian T. (2019). 3D Bioprinted Scaffolds Containing Viable Macrophages and Antibiotics Promote Clearance of *Staphylococcus aureus* Craniotomy-Associated Biofilm Infection HHS Public Access. ACS Appl. Mater. Interfaces.

[70] Mai H.N., Hyun D.C., Park J.H., Kim D.Y., Lee S.M., Lee D.H. (2020). Antibacterial Drug-Release Polydimethylsiloxane Coating for 3d-Printing Dental Polymer: Surface Alterations and Antimicrobial Effects. Pharmaceuticals.

